# Establishing a xenograft mouse model of peritoneal dissemination of gastric cancer with organ invasion and fibrosis

**DOI:** 10.1186/s12885-016-2991-9

**Published:** 2017-01-05

**Authors:** Mitsuyoshi Okazaki, Sachio Fushida, Shinichi Harada, Tomoya Tsukada, Jun Kinoshita, Katsunobu Oyama, Tomoharu Miyashita, Itasu Ninomiya, Tetsuo Ohta

**Affiliations:** 1 Department of Gastroenterological Surgery, Division of Cancer medicine, Graduate School of Medical Science, Kanazawa University, 13-1 Takara-machi, Kanazawa, 920-8641 Ishikawa Japan; 2 Center for Biomedical Research and Education, School of Medicine, Kanazawa University, 13-1 Takara-machi, Kanazawa, 920-8641 Ishikawa Japan

**Keywords:** Gastric cancer, Peritoneal dissemination, Organ invasion, Fibrosis

## Abstract

**Background:**

The clinical prognosis of gastric cancer with peritoneal dissemination is poor because of its chemoresistance and rich fibrosis. While several gastric cancer cell lines have been used to establish models of peritoneal dissemination by intraperitoneal injection, most peritoneal tumors that form adopt a medullary pattern in microscopic appearance. This histological finding for the model differs from that in the clinical situation. This study was performed to demonstrate the contribution of human peritoneal mesothelial cells (HPMCs) to fibrotic tumor formation and to establish a new xenograft model with high potential for peritoneal dissemination with organ invasion and extensive fibrosis.

**Methods:**

We established four types of xenograft model: i) intraperitoneal injection of MKN45-P cells alone (control group), ii) injection of MKN45-P cells co-cultured with HPMCs (co-cultured group), iii) scratching the parietal peritoneum (parietal group), and iv) scratching the visceral peritoneum (visceral group) with a cotton swab before injection of co-cultured cells. Fibrosis, α-smooth muscle actin expression, and organ invasion by tumor cells were all assessed by immunohistochemical examination.

**Results:**

All mice developed abdominal swelling with peritoneal tumors and bloody ascites. Tumors of the control and co-cultured groups were not invasive or fibrotic. Contrastingly, tumors of the scratch groups exhibited rich stromal fibrosis and possessed increased α-smooth muscle actin (α-SMA) expression. In particular, the visceral group showed edematous and spreading tumors invading the intestinal wall.

**Conclusion:**

We established a model of peritoneal dissemination with organ invasion and stromal fibrosis. Formation of peritoneal dissemination required a favorable environment for cell adhesion, invasion, and growth. This model may be useful for analyzing the pathogenesis and treatment of peritoneal dissemination of gastric cancer.

**Electronic supplementary material:**

The online version of this article (doi:10.1186/s12885-016-2991-9) contains supplementary material, which is available to authorized users.

## Background

Gastric cancer is one of the most common malignant diseases worldwide [1]. Peritoneal dissemination is a characteristic feature of gastric cancer and is a critical factor underlying its poor prognosis [2–4]. While clinical outcomes for gastric cancer patients with peritoneal dissemination have improved with advances in systemic and/or intraperitoneal (i.p) chemotherapy, desirable outcomes remain elusive [5–10]. Peritoneal dissemination is characterized by cancer cell infiltration and proliferation accompanied by extensive stromal fibrosis [11, 12]. This results in the development of chemoresistance and obstructive disorders such as ileus, obstructive jaundice, and hydronephrosis. Therefore, control of organ invasion and fibrosis is required to improve outcomes for patients with gastric cancer.

While several gastric cancer cell lines undergo peritoneal dissemination following intra-peritoneal injection in mice, almost all peritoneal tumors formed in this manner adopt a medullary pattern [13–15]. We have previously reported that fibrotic tumors can be established in a subcutaneous xenograft model using the gastric cancer cell line MKN45 in co-culture with human peritoneal mesothelial cells (HPMCs) [16]. However, a xenograft model of peritoneal dissemination is required, as delivery of therapeutic agents to the peritoneal cavity is limited following intravenous administration because of the peritoneal-blood barrier [17]. The establishment and analysis of a xenograft model that exhibits similar histopathological features to tumors from human gastric cancer patients with peritoneal dissemination may facilitate the development of treatments for this disease.

Therefore, this study aims to establish a new xenograft model of peritoneal dissemination with organ invasion and fibrosis akin to the typical clinical situation.

## Methods

### Patients and cell lines

Surgical specimens of human omentum were obtained from patients with no evidence of peritoneal inflammation and/or malignancy who underwent surgery in Kanazawa University Hospital between April and December 2013. Donors were not subjected to chemotherapy or radiation treatment prior to surgery. All patients provided written informed consent prior to participation in the study. The study was approved by the Research Ethics Committee of Kanazawa University (permission number 1747). HPMCs were isolated from surgical specimens of human omentum as previously described [18]. Briefly, small pieces of omentum were surgically resected under sterile conditions and were incubated in pre-warmed phosphate-buffered solution (PBS) containing 0.125% trypsin/EDTA (Gibco/Invitrogen, USA) for 30 min at 37 °C. The suspension was centrifuged at 1500 × *g* for 5 min. Collected cells were cultured in RPMI-1640 medium (Gibco/Invitrogen) supplemented with 20% heat-inactivated fetal bovine serum (FBS; Nichirei Bioscience Inc., Japan). The cells were cultured at 37 °C in a humidified atmosphere of 5% CO2 in air. For the following experiments, cells were used during the second or third passage after primary culture. HPMCs possibly contaminated with endothelial cells or fibroblasts at the time of harvest were not used. We used homogeneous HPMCs from a different donor for each experiment. We used the high-potential peritoneal dissemination cell line MKN45-P, which was established from the MKN45 gastric cancer cell line (Additional file 1: Figure S1) in our institution as described previously [19]. Briefly, female immunocompromised BALB/c-nu/nu mice (Charles River Laboratories Inc. Japan) were subcutaneously inoculated with MKN45 cells and the subcutaneous nodules were removed and injected into other mice intraperitoneally. The cancer cells from peritoneal nodules were injected into the abdominal cavity of other mice. The process was continued through to a seventh generation. The resulting cell line was named MKN45-P. Cells were maintained in RPMI-1640 medium supplemented with 10% FBS.

### Mouse xenograft model

**Fig. 1:**
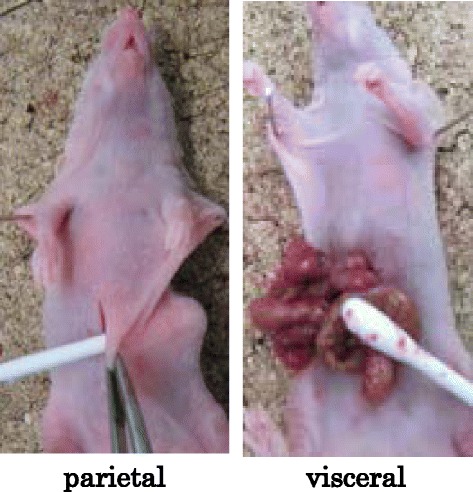
Methods of peritoneal scratching in the xenograft model. Representative images demonstrating the procedure of scratching the peritoneum with a cotton swab. *Left panel*: scratching of the parietal peritoneum. *Right panel*: scratching of the visceral peritoneum

All animal experiments were performed according to Kanazawa University’s standard guidelines. Female immunocompromised BALB/c-nu/nu mice at 4–6 weeks of age were maintained in a sterile environment. MKN45-P cells were co-cultured with an equivalent number of normal HPMCs for 5 days, and a total of 5 × 10^6^ cells in 1000 μL of RPMI-1640 were then i.p. injected into nude mice on day 0 (co-cultured group). For the control group, 5 × 10^6^ MKN45-P cells alone were injected (control group). The scratch method of i.p. cell inoculation was employed. First, an abdominal incision was made under general diethyl-ether inhalation anesthesia. For the parietal peritoneum scratch model, the left ventral parietal peritoneum was scratched using a cotton swab (Fig. 1a, parietal group). For the visceral peritoneum scratch model, the intestinal tract and mesenterium was removed from the peritoneal cavity and scratched using a cotton swab (Fig. 1b, visceral group). The visceral were then returned into the peritoneal cavity and the abdominal wall was closed. Next, a total of 5 × 10^6^ cells of a MKN45-P and HPMCs co-culture was injected i.p. and animals were carefully monitored. After 14 days, mice were anesthetized with diethyl-ether, sacrificed, and the tumors and abdominal organs excised together. Tumor specimens were then collected for immunohistochemical examination.

### Immunohistochemistry

Tumor specimens were fixed in 10% neutral buffered formalin and embedded in paraffin. Sections were stained with hematoxylin and eosin (H&E) and Azan stain for assessment of fibrosis, while the expression of α-smooth muscle actin (α-SMA; 1A4, mouse monoclonal IgG, diluted 1:100; Dako Cytomation, Denmark) and vimentin (V9, mouse monoclonal IgG, diluted 1:100; Santa Cruz Biotechnology, Inc.) was also assessed immunohistochemically. Deparaffinized sections were pretreated by autoclaving in 10% citric acid buffer (pH 8.0) at 120 °C for 15 min. Following treatment with protein block serum (Dako Cytomation, Kyoto, Japan) for 10 min and incubation with 2% skim milk for 30 min to block non-specific reactions, sections were incubated with primary antibody at 4 °C overnight. The Envision-polymer solution (horseradish peroxidase, HRP, Dako Cytomation) was then applied for 1 h. Signals were developed in 0.02% 3,3′-diaminobenzidinetetrahydrochloride solution containing 0.1% H_2_O_2_. Sections were then lightly counter stained with hematoxylin and examined using a fluorescence microscope (Olympus, Tokyo, Japan). The degree of fibrosis was calculated as a percentage of fibrosis within the whole section in all samples using a BZ-9000 BZII microscope (Keyence, Osaka, Japan).

### Statistical analysis

Differences among the data sets were evaluated using one-way analysis of variance or two-sided Student’s *t*-tests with the computer software package SPSS 10.0 (SPSS, Chicago, IL, USA). P values less than 0.05 indicated a statistically significant difference.

## Results

### Macroscopic appearance

**Fig. 2:**
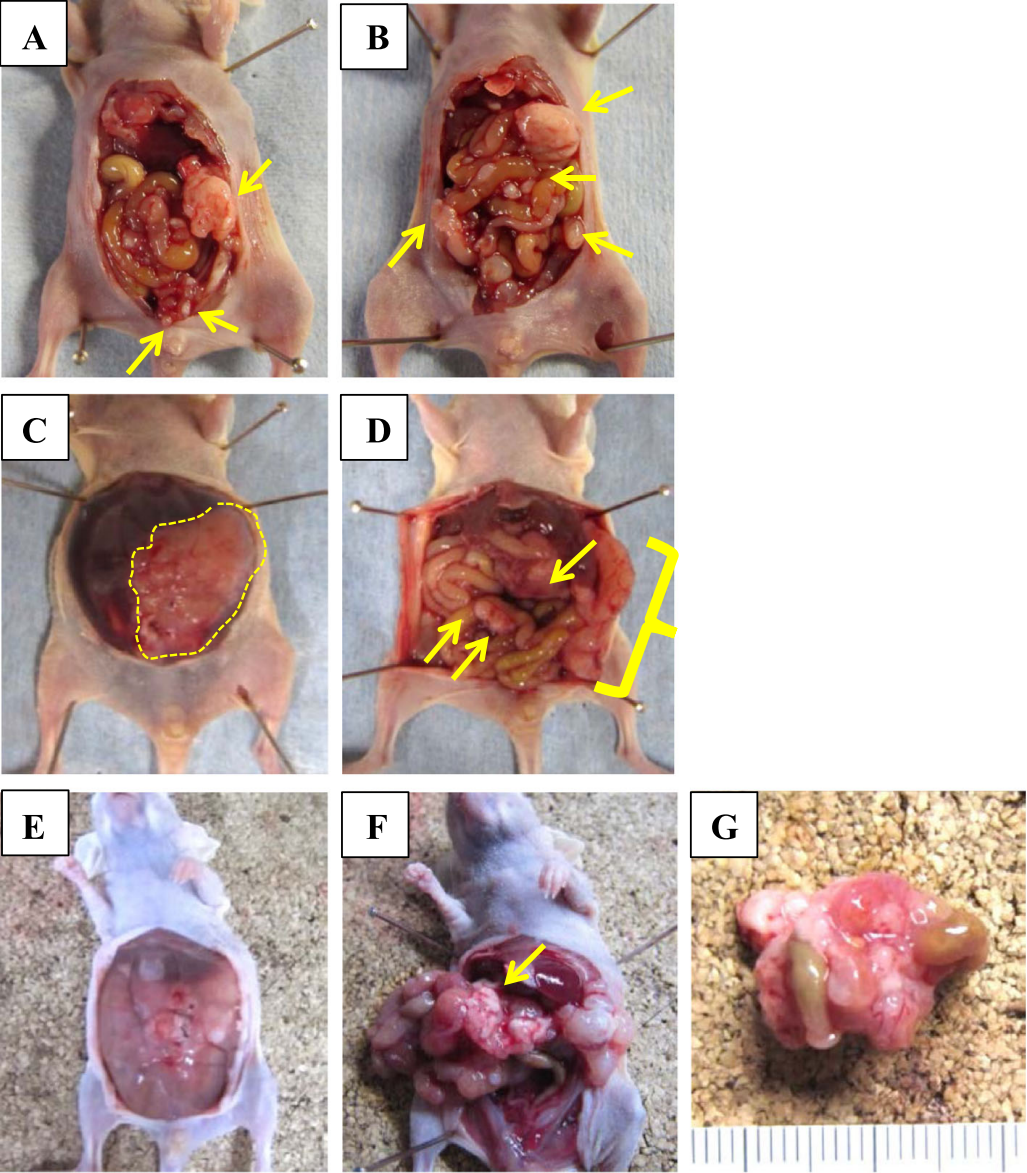
Representative images depicting four patterns of xenograft models at day 14. Macroscopic views of peritoneal nodules (*arrow head*). **a** control group. **b** co-cultured group. Peritoneal nodules are present on the surfaces of the abdominal organs, but organ invasion is not present. **c**, **d** parietal group. Tumors are present on the scratched peritoneal portion (Dotted line). **e**–**g** visceral group. Tumors forming a block with invaded intestinal tract and peritoneum

Representative images depicting the macroscopic appearance of the tumors at Day 14 are shown in Fig. 2. All mice developed abdominal swelling with peritoneal tumors and bloody ascites. Tumors of both control and co-cultured groups underwent peritoneal dissemination, though this occurred on abdominal organ surfaces and organ invasion was not observed (Fig. 2a, b). Mice of the parietal group had tumors present on the left side of the peritoneal cavity at the scratch sites (Fig. 2c). However, dissemination nodules did not exhibit organ invasion (Fig. 2d). Dissemination nodules with adhesion and invasion to the intestinal tract, block formation, and distention and edema of the intestinal tract were observed in mice of the visceral group (Fig. 2e–g).

### Microscopic appearance

**Fig. 3:**
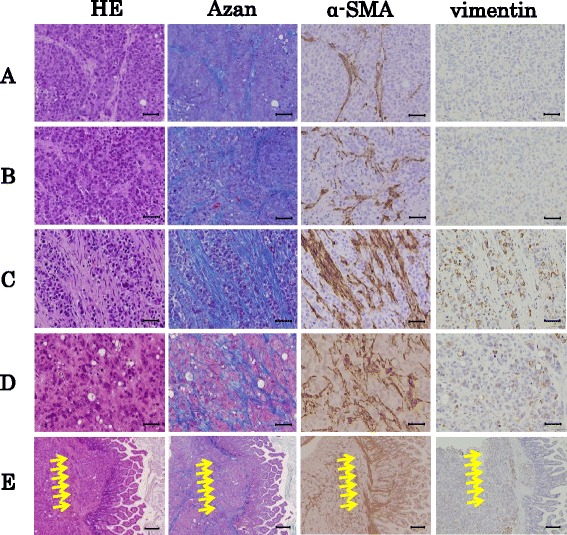
Histological examination of peritoneal dissemination xenograft models. Histological examination was performed by hematoxylin and eosin (*H&E*) staining, Azan, α-SMA *and vimentin* staining in the tumor. **a** control group, **b** co-cultured group, **c** parietal group, **d** and **e** visceral group. Tumors invaded to the intestine wall (*Arrows*). **a**, **b**, **c**, **d** original magnifications × 200. *Scale bar, 50 μm*. **e** × 40. *Scale bar, 200 μm*

**Fig. 4:**
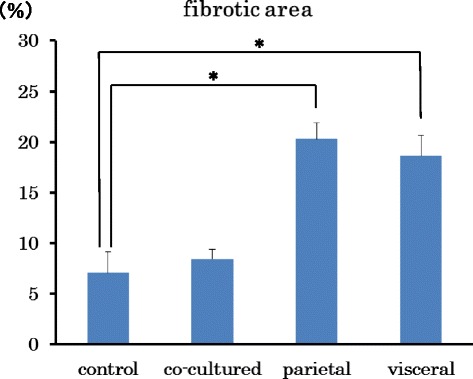
Fibrotic change in four patterns of xenograft model. Fibrotic areas were measured using Azan staining and are shown as a percentage (*fibrotic area*/*whole section area*). Data presented are the mean ± SD. *p < 0.05 versus control group

Control group tumors did not exhibit fibrosis or α-SMA and vimentin positive cells (Fig. 3a). Additionally, co-culture group tumors showed little fibrotic change (Fig. 3b). However, both the parietal and visceral peritoneum scratch model groups showed rich fibrosis and α-SMA positive cells (Fig. 3c, d). Furthermore, visceral group tumors invaded the intestinal tract by destroying the serosal membrane, as serosal edges stained positively for Azan and α-SMA (Fig. 3e). The scratch model group also exhibited cytoplasmic expression of vimentin. The fibrotic areas of tumors formed in conjunction with the scratch methods were significantly larger than those of control tumors (Fig. 4).

## Discussion

The peritoneal dissemination xenograft model described here mimics the clinical situation present in human patients in several respects. For example, bloody ascites and edematous intestines were present in the peritoneal cavity, tumors invaded surrounding tissues such as the digestive tract, and apparent stromal fibrosis was present. This is the first report to describe the successful establishment of a peritoneal dissemination model with organ invasion and fibrosis.

Previous studies have described peritoneal dissemination xenograft models that are based on the intraperitoneal injection of various gastric cancer cells into nude mice [13–15]. These models develop multiple nodules on the surfaces of abdominal organs and the peritoneal cavity. However, these tumors did not exhibit organ invasion or extensive stromal fibrosis. Other studies suggest that the volume and composition of fibrous tissue in various organs are influenced by epithelial-mesenchymal transition (EMT), which differentiate into an extracellular matrix-producing myofibroblast characteristics [20–22]. We have previously reported that transforming growth factor-β1 (TGF-β1) mediated activation of HPMCs induces an EMT-like process, and that activated HPMCs function as a source of cancer-associated fibroblasts [16, 23]. Furthermore, we have established a subcutaneous tumor model using the gastric cancer cell line MKN45 in co-culture with HPMCs [16, 24].

This study found that intraperitoneal injection of MKN45-P cells in co-culture with HPMCs did not result in formation of a fibrotic tumor with organ invasion. The MKN45-P cell line has a high potential for peritoneal dissemination and could adhere to the mouse peritoneal cavity; however, injected HPMCs could not adhere to the intact peritoneal surface. HPMCs are classified as epithelial in the broadest sense of the term, and serve as a protective anatomical barrier [25]. Yashiro et al. have previously demonstrated that a layer of confluent, intact mesothelial cells hindered cancer cell invasion to the abdominal cavity [16]. However, the presence of HPMCs stimulates a change in the complement of growth factors secreted by cancer cells [12, 16]. Therefore, the establishment of fibrotic tumors necessitates creation of a favorable environment within the abdominal cavity that enables both gastric cancer cells and HPMCs to grow.

Clinical experience suggests that patients with free cancer cells in the abdominal cavity do not necessarily develop peritoneal implantation. Over 100 years ago, Paget et al. proposed the ‘seed and soil’ theory: metastasis only occurs when tumor cells (seeds) survive and grow in a favorable organ/tissue microenvironment (soil) [26]. This theory is central to the formation of peritoneal dissemination in gastric cancer. For example, EMT of HPMCs in combination with peritoneal fibrosis provides a favorable environment for the dissemination of gastric cancer through the naked areas of a basal membrane [16, 19, 27]. TGF-β1 is considered a master switch for the induction of fibrosis during EMT in multiple organs and tissues, including the peritoneum [19–22, 28, 29]. In a clinical scenario of peritoneal dissemination, HPMCs would expose a basal membrane as an anchorage point by adopting a spindle-shape morphology under the influence of cancer cell-derived cytokines, including TGF-β1 [16]. Integrin molecules expressed on cancer cells play a crucial role in initial adhesion to the basal membrane [30, 31]. Later, cancer cell-derived matrix metalloproteinases degrade the basal membrane, facilitating cancer cell invasion of the deeper tissue [32]. Therefore, scratching of the mouse peritoneum likely facilitated formation of fibrotic and invasive tumors because the basal membrane was exposed as an anchorage site for cancer cells and HPMCs.

Peritoneal dissemination is the most frequent pattern of recurrence after curative resection of gastric cancer. Previous studies reported that peritoneal recurrence was evident in 43–57% of gastric cancer patients, while hematogenous recurrence occurred in 26–54%, and lymph node recurrence and local recurrence developed in 17–25.8% of individuals [33–36]. Our xenograft model mimics the peritoneal defect formed by the peeling of mesothelial cells during gastrectomy and lymph node dissection. In other words, surgical procedures themselves may provide a favorable environment for peritoneal dissemination. Therefore, future investigations should focus on the pathogenesis of peritoneal dissemination as well as novel treatment strategies for the prevention of peritoneal fibrosis.

## Conclusion

We have established a murine model of peritoneal dissemination that mimics the clinical findings of fibrosis and invasion in human gastric cancer patients. This model involves scratching of the peritoneal surface and injection of co-cultured gastric cancer cells and HPMCs. This model may be useful for evaluating strategies for treatment of peritoneal dissemination of gastric cancer.

## Additional file

Additional file 1: Figure S1. Cell line data sheet. (JPG 504 kb)

## Abbreviations

EMT: Epithelial mesenchymal transition
HPMCs: Human peritoneal mesothelial cells HPMCs
TGF-β: Transforming growth factor-β
α-SMA: α- Smooth muscle actin
