# Supplementary figures and images for: Establishing a xenograft mouse model of peritoneal dissemination of gastric cancer with organ invasion and fibrosis
Source: BMC Cancer. 2017 Jan 5;17:23. doi: 10.1186/s12885-016-2991-9 (PMC5217597; doi:10.1186/s12885-016-2991-9)

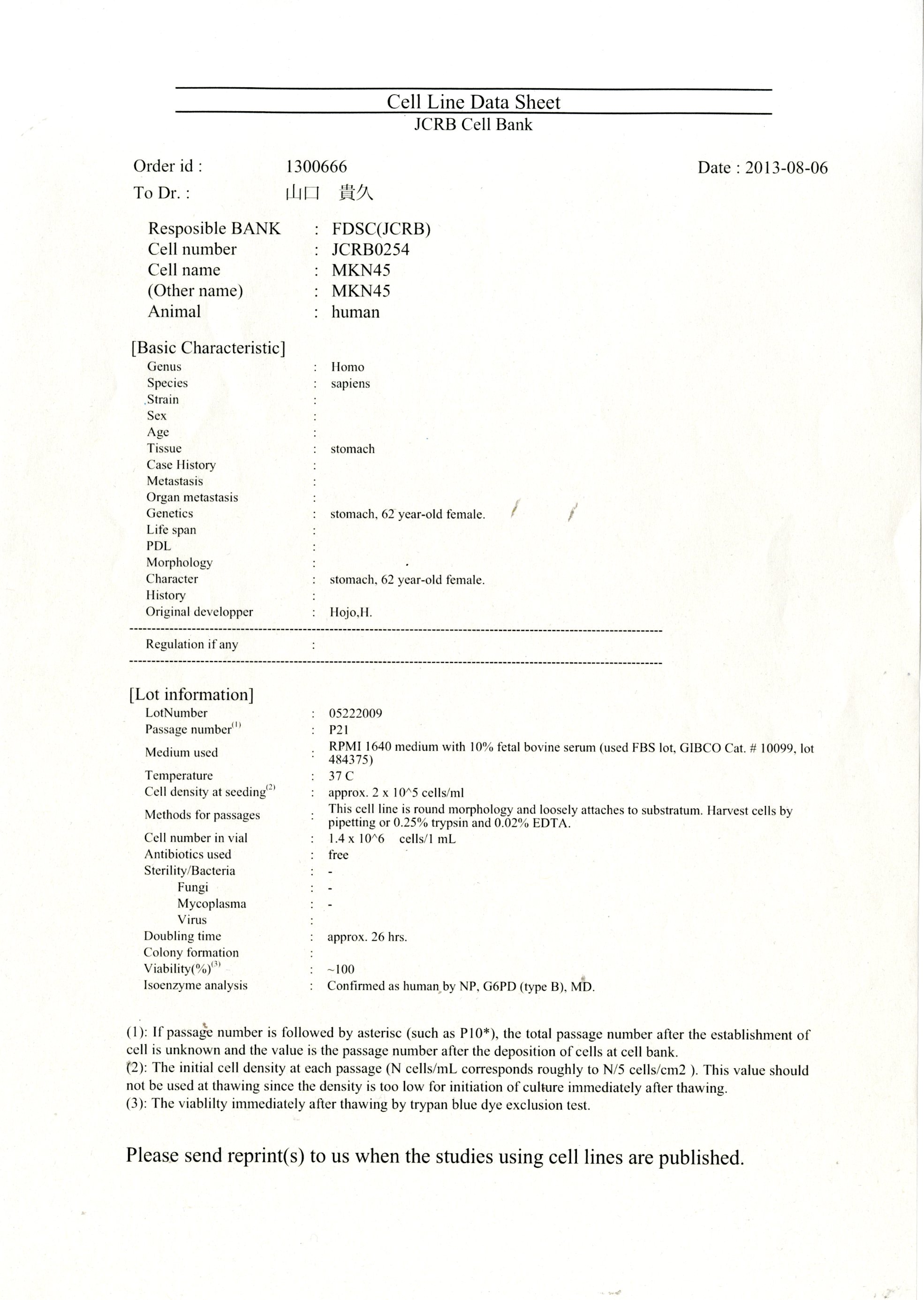

Supplement: Additional file 1: Figure S1. — Cell line data sheet. (JPG 504 kb) [file 12885_2016_2991_MOESM1_ESM.jpg]
